# Non-Destructive Determination of Bayberry Sugar and Acidity by Hyperspectral Remote Sensing of Si-Sensor and Low-Cost Portable Instrument Development

**DOI:** 10.3390/s23249822

**Published:** 2023-12-14

**Authors:** Jiaoru Wang, Weizhi Wu, Shoupeng Tian, Yadong He, Yun Huang, Fumin Wang, Yao Zhang

**Affiliations:** 1 College of Artificial Intelligence, Hangzhou Dianzi University, Hangzhou 310018, China; wangjiaoru923@163.com (J.W.); wwz123@hdu.edu.cn (W.W.); tianshoupeng@hdu.edu (S.T.); hyd123@hdu.edu.cn (Y.H.); 2 Jinhua Agricultural Science Study Institute, Jinhua 321000, China; huangyun_123@jhnky.cn; 3 Zhejiang Agricultural Machinery Research Institute, Jinhua 321000, China; 4 Institute of Agricultural Remote Sensing & Information Application, Zhejiang University, Hangzhou 310058, China; wfm@zju.edu.cn

**Keywords:** bayberry (*Myrica rubra cv. DongKui Orient Pearl*), sugar percentage and acidity, hyperspectral detection, low-cost portable instrument

## Abstract

The digitalization of information is crucial for the upgrading of the bayberry digital agriculture industry, while the low-cost information detection sensing equipment for bayberry are a bottleneck for the digital development of the industry. The existing rapid and non-destructive detection devices for fruit acidity and sugar content mainly use near-infrared and mid-infrared spectral characteristic for detection. These devices use expensive InGaAs sensor, which are difficult to promote and apply in the bayberry digital industry. This study is based on the high-spectral range of 454–998 nm in bayberry fruit to study the mechanism of fruit sugar and acidity content detection and to develop a portable bayberry fruit sugar and acidity detection device using Si-sensor in order to achieve low-cost quality parameter detection of bayberry fruit. The research results show that: Based on the hyperspectral of bayberry fruit, the sensitive wavelength for sugar content inversion is 610 nm, and the inversion accuracy (RMSE) is 1.399Brix; the sensitive wavelength for pH inversion is 570 nm, and the inversion accuracy (RMSE) is 0.1329. Based on the above spectroscopic detection mechanism and spectral dimension reduction methods, combined with low-cost Si-sensor (400–1000 nm), a low-cost non-destructive portable bayberry fruit sugar and acidity detection device has been developed, with detection accuracies of 94.74% and 97.14%, respectively. This bayberry fruit sugar and acidity detector provides a low-cost portable non-destructive quality detection instrument of bayberry, which is in line with the industrial group of low consumption in which the bayberry is mainly cultivated on a small scale, accelerating the digitalization process of the bayberry industry.

## 1. Introduction

Bayberry (*Myrica rubra cv. DongKui Orient Pearl*) is a special economic crop in South China. In Xianju county, Zhejiang province, China alone, the total output of bayberry reached 90,000 tons in 2019, with a total output value of $1.65 billion [1,2]. Bayberry fruit contains a variety of organic acids which can increase the acidity in the intestines and stomach and help digestion and absorption. Anthocyanins and vitamin C contained in this fruit have an antioxidant function, can improve immunity, and delay aging [3]. The fruit acid it contains prevents the conversion of sugar into fat in the body and helps with weight loss [4]. The traditional grade detection process mainly relies on manual sorting based on the color of bayberry fruit into different grades with the naked eye. According to empirical methods, the darker the color, the higher the sugar content and the lower the acidity [5].

Nonetheless, this method has high requirements for workers’ proficiency, strong subjectivity, and low sorting efficiency, which cannot meet the current digital demand of bayberry grade detection. At present, non-destructive testing instruments for fruits on the market, such as the K-SS900LC detector from Japan, the F-750 detector from the United States, and the TPF-750 detector from China, are commonly used in the near-infrared band for testing, using expensive indium gallium arsenide photodetectors (InGaAs sensor) [6,7]. Also, these instruments do not contain the specified model for detecting the sugar and acidity of bayberry. Therefore, it is difficult to widely promote them in small-scale bayberry planters. Additionally, bayberry fruits have a high moisture content, and the spectral information in the near-infrared band is susceptible to moisture interference, resulting in lower detection accuracy [8]. At present, PAL-1 sugar meter with an accuracy of ±0.2%, a destructive testing instrument, is commonly used to detect the sugar content of bayberry [9], and this method was employed for the validated data collection. Therefore, there is an urgent need to develop a non-destructive and fast detection instrument for bayberry fruit sugar and acidity content based on visible hyperspectral.

With the development of hyperspectral technology, the detection methods of manually identifying fruit variety quality and chemical analysis of fruit internal and external quality are being gradually replaced by hyperspectral non-destructive testing technology [10,11]. Shao [12] proposed a comprehensive prediction method for the acidity value of bayberry juice by combining visible/near-infrared spectroscopy with Partial Least Squares (PLS) and Artificial Neural Network (ANN) (sensitive wavelength range of 945–965 nm). Zhao et al. [13]. established a Partial Least Squares prediction model for wavelength and green plum pH value based on hyperspectral imaging using different dimension reduction and filtering methods (sensitive wavelength range of 556–843 nm). Extensive research has shown that hyperspectral non-destructive testing technology has been widely applied. Building upon this, we conducted a study on the non-destructive detection of sugar and acidity content in bayberries based on hyperspectral remote sensing in the visible light region. Common silicon photodetection devices (Si-sensor) are valued at just a few dollars, reducing the cost of instruments for visible light band detection. Moreover, numerous previous studies have shown a certain correlation between anthocyanins, sugar content and pH [14]. Cheng et al. [15]. using various apples as samples, demonstrated a significant correlation between the anthocyanin content of apple peels and pH during maturation. Additionally, in Mutsu apples, anthocyanins were significantly correlated with soluble solids, confirming the feasibility of using anthocyanins for sugar content inversion. Liu et al. [16] proposed a method for inverting the relative content of anthocyanins in corn leaves using hyperspectral remote sensing technology. The quadratic models based on RI (521, 698) and other models for anthocyanin inversion showed high coefficients of determination, indicating the feasibility of inversion anthocyanins through spectral technology.

Based on the above conclusions, this study proposes a non-destructive detection method for bayberry fruit sugar content and acidity based on visible hyperspectral information and conducts research on low-cost instrument development methods. On this basis, a low-cost non-destructive portable bayberry sugar content and acidity detection instrument is developed. This instrument uses a Si-sensor and a bandpass filter with a bandwidth of 32 nm, priced at around $100, which is far cheaper than the nearly $1000 portable lossy sugar meter (such as PAL-1) on the market. This enables individual farmers to conduct classification detection and packaging, differing from the previous situation where only large enterprises could perform classification detection. This has significant implications for simplifying bayberry production processes and improving overall economic income.

## 2. Acquisition and Preprocessing of Data

The experimental samples in this article were bayberry collected from a bayberry picking base in Xianju, Zhejiang province on 21 June 2020. The bayberry was divided into four grades through skilled sorting workers in the local area. Two hundred bayberries of uniform shape and size and no extruded water were selected, fifty of each grade, and the sample numbers given were 1–200. Hyperspectral images of each bayberry were collected under a natural light environment. Anthocyanin content, total sugar, soluble solids, and pH value were measured in a laboratory environment.

### 2.1. Acquisition and Processing of Hyperspectral Image Data of Bayberry Fruit

The UHD-185 hyperspectral imager was used to collect hyperspectral images of bayberry under natural light conditions in the field. The UHD-185 hyperspectral imager has a spectral resolution of 4 nm, a sampling interval of less than 100 μs, and a spatial resolution of 1mm within the wavelength range of 454 nm to 998 nm [17]. Hyperspectral images were collected using the Spectralon^®^ (North Sutton, NH, USA) standard diffuse reflectance whiteboard before measurement to calibrate the hyperspectral images and a tripod was used to allow the UHD-185 to capture the hyperspectral images of bayberry in a fixed position. The following figure shows the spectral characteristics of four different grades of bayberry, and grade I is the optimal one.

**Figure 1:**
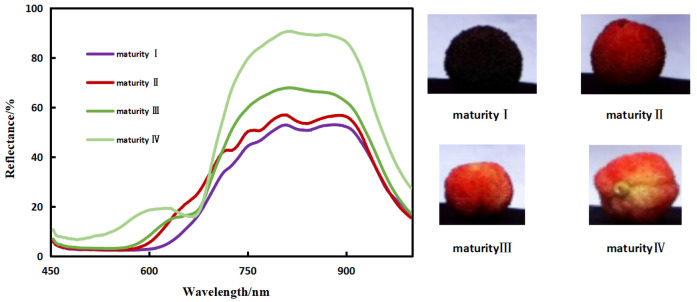
Bayberry spectra with different grades.

According to the spectral characteristics in Figure 1, the reflectance of different grades of bayberry significantly changes in the 530–630 nm wavelength range, and the reflectance decreases with quality improvement. Therefore, this range is determined to be the sensitive band for bayberry quality.

### 2.2. Determination of Physical and Chemical Data of Bayberry Fruit

In order to model the sugar and acidity of bayberry, this article conducted experimental measurements on the anthocyanin content, total sugar content, soluble solids, and pH value of bayberry fruit.

This article uses spectrophotometry to measure the content of anthocyanins in bayberry [18]. Anthocyanin extraction solution was prepared in a ratio of 2:50 between bayberry and solution [19,20]. This article uses the anthrone-sulfuric acid method to measure the total sugar content of bayberry [21]. The sample solution was prepared in a ratio of 2:3000 between bayberry and solution, and the measurement unit is μg/g. This article uses the PAL-1 digital sugar meter developed by the Atago Company in Japan (Fukaya-shi, Japan) to measure the soluble solids in bayberry (the actual measurement data of the sugar meter is soluble solids, but when there are fewer other contents and more sugar content in the solution, the measurement result can be considered as sugar content [22]), the accuracy of this instrument is ±0.2% [23]. A bayberry solution was prepared in a ratio of 2:10 between bayberry and solution to measure the sugar content and conversion to obtain the sugar content of the bayberry; the unit is Brix. For the pH value of waxberry, we use a pH meter to measure it. A bayberry solution was prepared in a ratio of 2:1000 between bayberry and solution to measure the pH value of the bayberry solution and convert it to obtain the pH of the bayberry.

### 2.3. Componpents Characteristics of Bayberry Grade

As shown in Table 1, there is a significant difference between the maximum and minimum values of anthocyanins in bayberry, with 518.04 μg/g and 10.18 μg/g, indicating a significant difference in anthocyanin content among different grades of bayberry. The standard deviation of the pH value determination is only 0.21, indicating that there have been no significant changes in the pH fluctuation among different grades of bayberry. The sugar content fluctuates relatively closely. The measured sugar content, acidity, and anthocyanin content are evenly distributed, covering various grades of bayberry, which meets the subsequent modeling requirements.

## 3. Results and Discussion

### 3.1. Spectral Non-Destructive Detecting Mechanism of Sugar and Acidity in Bayberry Fruit

Traditional manual detection of the grade of bayberry mainly involves the human eye qualitatively dividing it into several grades based on color (visible light region), and the color of bayberry is determined by its anthocyanin content [24]. The study on inverting anthocyanin content through hyperspectral analysis is relatively mature [25,26]. This article attempts to find the relationship between anthocyanin content, sugar and acidity, and uses hyperspectral information to quantitatively detect the sugar and acidity of bayberry, achieving the datafication of bayberry grade sorting. By using visible light sensors instead of near-infrared and mid-infrared sensors, the cost of the instrument can be reduced.

#### 3.1.1. Analysis of the Relationship between Sugar and Acidity and Anthocyanin Content in Bayberry Fruit

The correlation between the total sugar, sugar content, and pH value measured and the anthocyanin content was analyzed.

**Figure 2:**
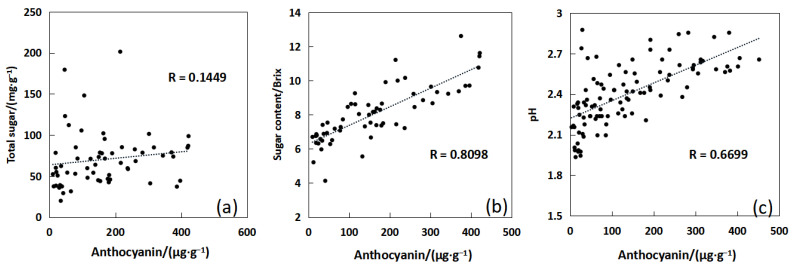
Correlation analysis between physical and chemical parameters of bayberry fruit and anthocyanin content. (**a**) Correlation between bayberry total sugar and anthocyanin content (**b**) Correlation between bayberry sugar content and anthocyanin content (**c**) Correlation analysis between bayberry fruit pH and anthocyanin content.

Figure 2 shows that there is a high correlation between sugar content and anthocyanin content, with a correlation coefficient of 0.8098. A relationship model between anthocyanin and sugar content can be constructed, and the sugar content of bayberry can be inverted using hyperspectral analysis. The correlation between total sugar and pH value with anthocyanins is relatively low, so it cannot be directly inverted through anthocyanin content.

#### 3.1.2. Analysis of the Relationship between the Displacement Characteristics of Anthocyanin Absorption Peak and pH Value in Bayberry Fruit

**Figure 3:**
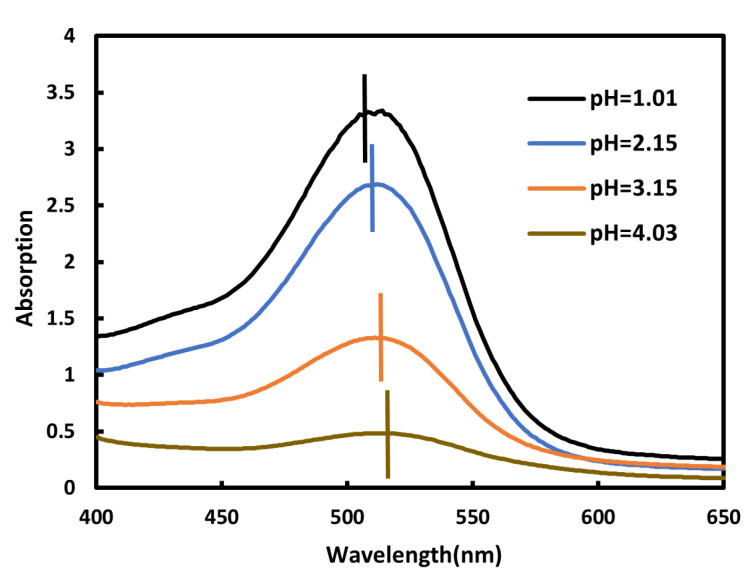
Absorption spectra of bayberry solution at different pH.

Figure 1 shows the spectral reflectance of four different grades of bayberry fruits at 454–998 nm. It can be concluded from this figure that the tendencies in spectral reflectance of different grades of bayberry fruits are basically consistent. There is a significant difference in spectral reflectance in the band of 530–630 nm, and its trend is consistent with the change in grade of bayberry fruit. The distinction between different grades in the bands after 750 nm is also relatively obvious, but the trend of spectral changes is inconsistent with the trend of grade changes. There is a reflectance peak between 550 and 650 nm, and this peak will decrease with the improvement of bayberry quality. In high-quality bayberries, this peak basically disappears. At the same time, in Figure 1, we can observe that the peak position will shift to the left as the quality of bayberry improves. The shift of anthocyanin absorption peak may be caused by changes in pH. This is a similar phenomenon in the PROSPECT-D of F é ret and J. B, where the absorption peak position of anthocyanins changes with pH value [27]. We conducted experiments on this and measured the absorption spectra of bayberry solutions adjusted to different pH values using hydrochloric acid and sodium hydroxide. Figure 3 shows that the absorption peak positions of bayberry solutions at different pH values make a significant shift, and as the pH value of the bayberry solution increases, the absorption peak of the bayberry solution absorption spectrum shows a significant right shift trend. There is a certain relationship between the pH values of the spectrum of bayberry. This indicates that it is feasible to establish a pH model for bayberry fruit using spectroscopy.

### 3.2. Construction and Validation of a Non-Destructive Detection Model for Sugar and Acidity of Bayberry Fruit

According to the above research and analysis, the characteristics of anthocyanins are linearly related or significantly consistent with sugar content and pH value in bayberry fruit. Based on band sensitivity analysis, this article establishes a spectral method that conforms to the inversion of sugar content and pH in bayberry fruit by the reflectance of the bayberry fruit at 530–630 nm and spectral normalization index. This article selects hyperspectral and physicochemical data from eighty bayberries to establish a model and validates the model with hyperspectral and physicochemical data from twenty bayberries. Constructing normalization index of spectrum NI [28], as shown in the following equation:

(1) $$NI=\frac{R_{i}-R_{j}}{R_{i}+R_{j}}$$

where $R_{i}$ and $R_{j}$ represents the reflectance of bayberry at the wavelength of i nm and j nm, respectively.

#### 3.2.1. Establishment of Inversion Model for Sugar and Acidity of Bayberry Fruit

**Figure 4:**
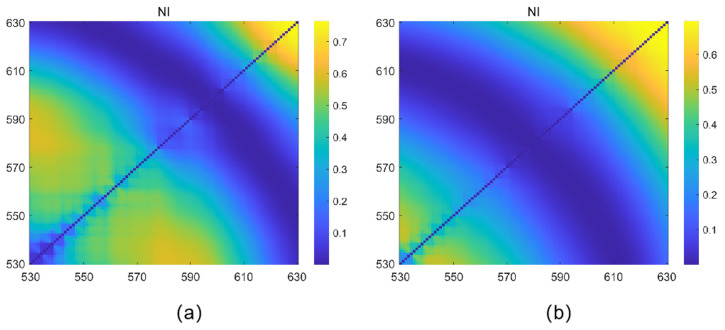
Correlation coefficient (*R*^2^) between the normalized index NI and the content of anthocyanins (**a**) and pH (**b**). The abscissa and ordinate indicate the wavelength in nm.

According to the analysis of spectral characteristics, the variation of reflectance with anthocyanin content is more significant in the 530–630 nm wavelength range. Calculate the normalization index of any combination of two bands within the 530–630 nm wavelength range and calculate the correlation coefficient $R^{2}$ between the normalization index and the anthocyanin content and pH value of bayberry, as shown in Figure 4. Through the analysis of correlation coefficients, the bands of normalized index NI were selected as 620 nm and 630 nm, and the correlation coefficient between normalized index NI and anthocyanins was relatively high in this band. The selected bands for normalization index NI are 620 nm and 630 nm, and the correlation coefficient between normalization index NI and pH value is relatively high in this band.

**Figure 5:**
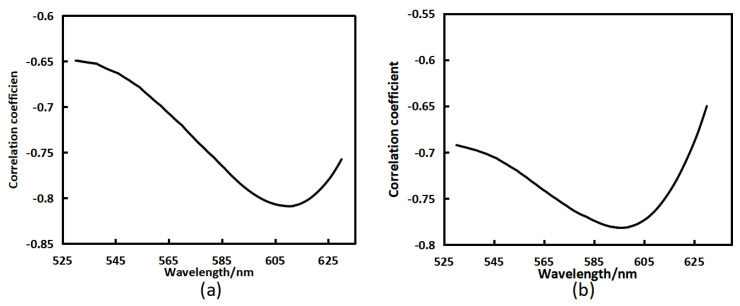
Correlation coefficient between bayberry reflectance and the content of anthocyanin (**a**) and pH (**b**).

Calculate the correlation coefficient $R^{2}$ between the reflectance and anthocyanin content of bayberry at 530–630 nm, as shown in Figure 5a. From Figure 5a, the highest correlation coefficient is 0.81 at 610 nm. At this band, the correlation between reflectance and anthocyanin content is highest. Therefore, the band of reflectance was selected as 610 nm. Calculate the correlation coefficient $R^{2}$ between the reflectance and pH value of bayberry at 530–630 nm, as shown in Figure 5b. From Figure 5b, the correlation coefficients are high at 560–620 nm. Considering the low-cost of subsequent instrument development, the reflectance band was selected as 570 nm for inversion.

Using the selected wavebands, the unitary linear relationship, the unitary quadratic relationship, the exponential relationship, the logarithmic relationship and the power function relationship between the normalized index NI, reflectance and anthocyanin content and pH value of bayberry were constructed, as shown in Table 2 below.

According to the correlation analysis, the $R^{2}$ of the univariate quadratic model for invert anthocyanin content using reflectance is 0.6795, and the $R^{2}$ of the univariate quadratic model for inverting anthocyanin content using normalized index NI is 0.6458. The correlation between the two is relatively close. However, due to considerations of practicality and portability in instrument production, the reflectivity at 610 nm was ultimately selected to invert the anthocyanin content of bayberry fruit. Its model is

(2) $$C_{anth}=0.7375R_{620}^{2}-36.21R_{620}+483.7$$

$C_{anth}$ is the anthocyanin content in the fruit of bayberry, in units of μg/g, $R_{610}$ is the reflectance of bayberry in the 610 nm band. The relationship between the anthocyanin content and the sugar content of bayberry was constructed, including the unitary linear relationship, the unitary quadratic relationship, the exponential relationship, the logarithmic relationship and the power function relationship. The determination coefficient $R^{2}$ of the unitary linear model for inverting sugar content using anthocyanins is 0.6658, indicating a high correlation. Therefore, the model for inverting sugar content using anthocyanins in bayberry fruit is selected, as shown in Equation (3).

(3) $$C_{sugar}=0.01087C_{anth}+6.284$$

In Equation (3), $C_{sugar}$ is the sugar content in the fruit of bayberry, in units of Brix. $C_{anth}$ is the content of anthocyanins in the fruit of bayberry.

The $R^{2}$ of the power function model for inversion of pH value using reflectance is 0.6589, and the $R^{2}$ of the unitary quadratic model for inversion of pH value using normalization index NI is 0.7071. Although the model of inverting pH value using normalization index performs well, considering the practicality and portability of instrument production, the reflectance at 570 nm was ultimately selected to invert the pH value of bayberry fruit. The model is shown in Equation (4).

(4) $$PH=1.515R_{570}^{-0.2733}+1.434$$

In Equation (4), PH is the pH value of the fruit of bayberry, $R_{570}$ is the reflectance of bayberry in the 570 nm band.

#### 3.2.2. Validation of the Sugar Acidity Inversion Model for Bayberry Fruit

**Figure 6:**
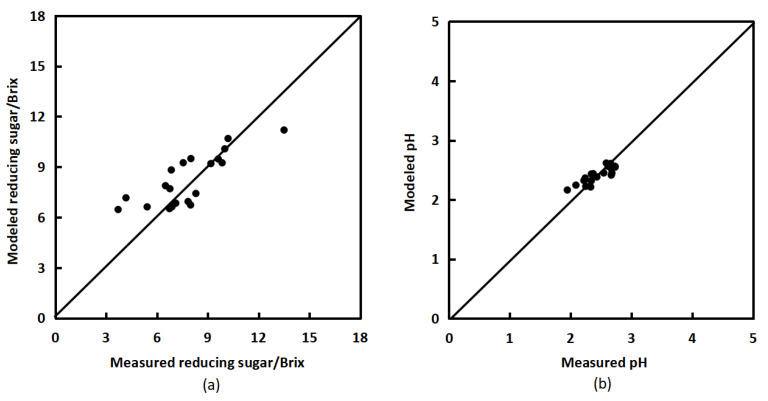
Comparison of modeled and measured values of reducing sugar content (**a**) and pH (**b**) in bayberry.

The anthocyanin content of twenty bayberries was extracted and measured using a methanol hydrochloric acid aqueous solution, and the sugar content and pH value of these twenty bayberries were measured using a sugar meter. The inversion model mentioned above was used to calculate the sugar content and pH value of bayberry, and this was compared with the measured values to verify the performance of the sugar content inversion model and pH value inversion model. The validation root mean square error (RMSE) of the sugar content inversion model was 1.399, and the validation root mean square error (RMSE) of the pH value inversion model was 0.1329, both reaching a high level. Figure 6a shows the comparison between simulated and measured values of sugar content. The two-dimensional scatter points composed of simulated and measured values of sugar content of bayberry are all roughly around the 1:1 line, indicating that the model for the relationship between anthocyanins and sugar content in the bayberry fruit is effective. Figure 6b shows the comparison between simulated and measured pH values. The two-dimensional scatter points composed of simulated and measured pH values of bayberry are all roughly around the 1:1 line, indicating that the acidity inversion model in bayberry fruit is effective.

### 3.3. Low-Cost Portable Bayberry Fruit Quality Testing Device

Based on the sugar acidity inversion model of bayberry, a low-cost sugar acidity detector for bayberry fruit was developed. This instrument is applied to quality detection in unnatural light source scenarios, using two characteristic wavelength LED lights as light sources; reflected light information on the surface of bayberry fruit was collected through filters and Si-sensor to further invert sugar and acidity.

#### 3.3.1. Consistency Analysis of Characteristic Spectrum and Inversion RESULT of Bayberry Sugar and Acidity at Different Spectral Resolutions

The spectral resolution of the spectral image reaches 4 nm, and each channel contains 4 nm of spectral data. It is necessary to install a filter in front of the lens of an industrial camera to remove the image information in bands other than characteristic bands. At present, the commonly used filter has a bandwidth of 30 nm, which cannot meet the requirement of 4 nm spectral resolution of spectral images. The characteristic bands of bayberry detection were analyzed, and the spectral information of the band was compared with a bandwidth of about 30 nm centered around the characteristic band with the spectral information of the 4 nm characteristic band in the spectral image. If they are relatively consistent, the characteristic bands can be expanded to 30 nm, thus reducing the cost of the instrument.

**Figure 7:**
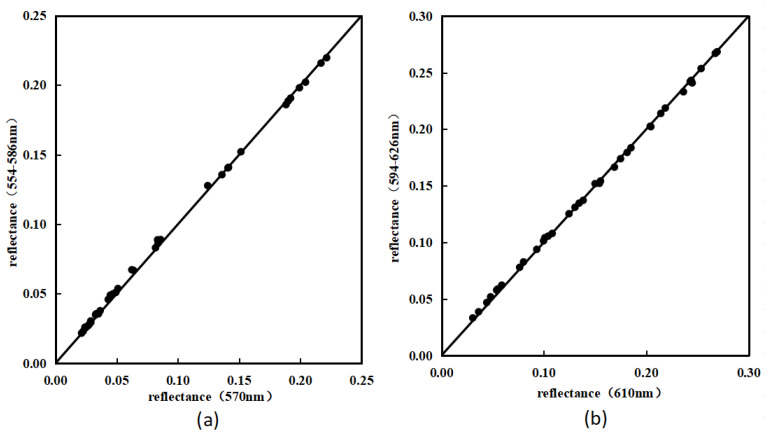
Comparison of bayberry spectrum at 570 nm and the average spectrum in 554–586 nm (**a**), Comparison of bayberry spectrum at 610 nm and the average spectrum in 594–626 nm (**b**).

The spectral resolution in the spectral image is high (4 nm), so the characteristic band is taken as the central wavelength, and 16 nm wavelength is extended to both sides as the expanded spectral resolution (32 nm), and the characteristic bands become 554–586 nm and 594–626 nm. Based on the spectral response characteristics of different quality bayberry, several sample spectra were selected from the corresponding spectral datasets, and the average spectra in the range of 554–586 nm and 594–626 nm were extracted from them; this was compared with the characteristic spectra of 570 nm and 610 nm. The obtained spectral data is shown in Figure 7.

From Figure 7, it can be observed that there is indeed some consistency between the 32 nm bandwidth spectrum and the characteristic spectrum of different quality samples of bayberry. The spectral reflectance of each sample is relatively close, and the RMSE values between the spectra of the 570 nm and 610 nm characteristic bands are 0.0023 and 0.0021, respectively, which meet the accuracy requirements. This indicates that the spectral response of bayberry has a high consistency near the two characteristic wavelengths. Therefore, it is feasible to use the spectral information with a 32 nm bandwidth as a substitute for the characteristic spectrum.

**Figure 8:**
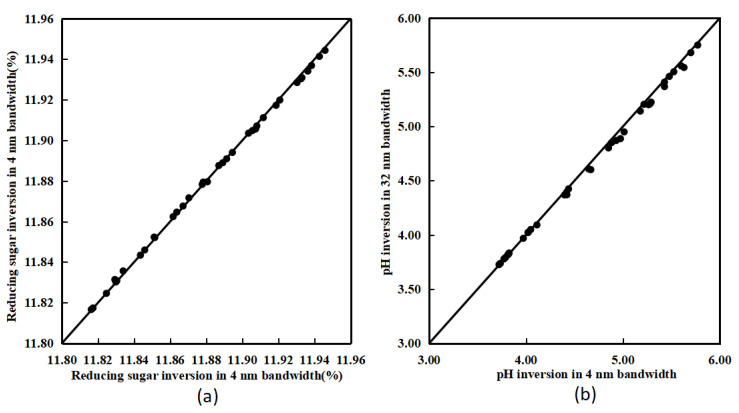
The pH inversion results with different bandwidth (**a**) and reducing sugar content inversion results with different bandwidth (**b**).

The characteristic spectra with different spectral resolutions have relatively consistent spectral information, but there are still some differences that can cause errors in the inversion of acidity and sugar content. Therefore, it is necessary to compare the inversion results of different bandwidth characteristic spectra for each sample to analyze whether the final error meets the accuracy requirements. Based on the acidity and sugar content inversion model mentioned earlier, the different inversion results for each sample are shown in Figure 8.

Similarly, the inversion results of the characteristic spectra with different spectral resolutions are also relatively consistent, indicating that the inversion error caused by spectral error will not affect the inversion of relevant chemical components. The RMSE values for pH value and sugar content inversion accuracy are 0.04 and 0.001, respectively, meeting the accuracy requirements. Therefore, the bayberry quality characteristic wavelengths of 570 nm and 610 nm based on spectral imaging can be extended to the 554–586 nm and 594–626 nm bands, respectively. The spectral information of different bandwidths has a high consistency in the inversion of sugar and acid content, which provides feasibility for the RGB sensor to acquire characteristic spectral information.

#### 3.3.2. Instrument Development Method

In this instrument, the light source adopts an LED controllable light source and collects illumination information through Si-sensor. It is necessary to record the illuminance of the light source as well as the illuminance of the light source after reflecting on the object to calculate the reflectance of an object. The illuminance reflected by an object can directly collect through Si-sensor, while the illuminance of the light source needs to be calibrated using a standard diffuse reflectance whiteboard according to the requirements of reflectivity collection. The calibration of LED light sources is performed by collecting the illuminance of the light source reflected by a standard whiteboard as its calibration value. Keeping the supply voltage of the LED unchanged, this calibration value can be used as a fixed parameter to calculate the reflectivity of different objects. The reflectivity of the measured object can be calculated through the following method:

(1) Take the calibration results of 570 nm and 610 nm light sources which is LED1 and LED2 as the incident intensities E (LED1) and E (LED2), respectively.
(2) Take the illuminance values of the two LED light sources reflected by the measured object collected by Si-sensor as the reflection intensity value L (LED1) and L (LED2), respectively.
(3) Calculate the reflectivity of the measured object at two wavelengths separately: *R*_570_ = L(LED1)/E(LED1), *R*_610_ = L(LED2)/E(LED2).

#### 3.3.3. Hardware Composition and Structure

**Figure 9:**
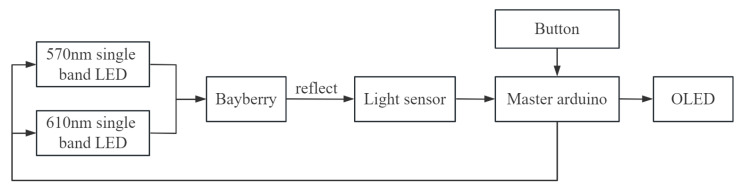
Portable bayberry quality detector overall hardware block diagram.

**Figure 10:**
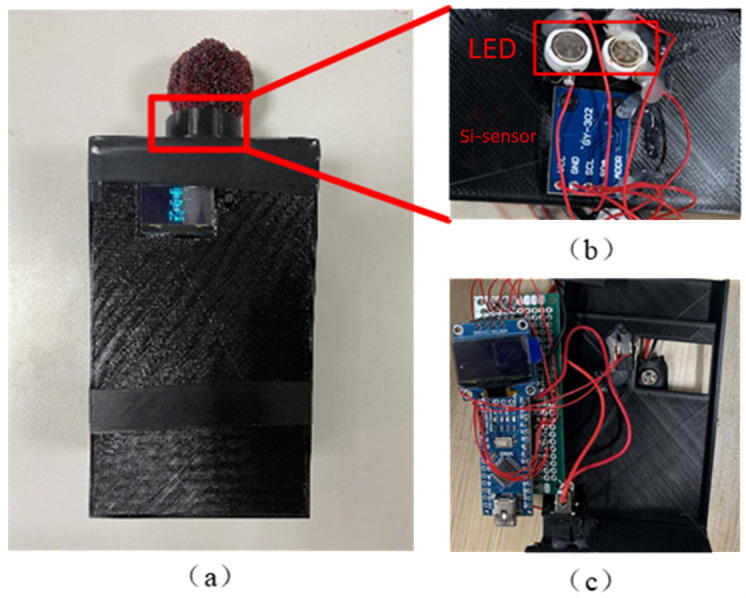
Schematic diagram of the portable bayberry quality detector ((**a**) Overall diagram of the instrument; (**b**) Light source and detection part; (**c**) Internal structure).

The portable quality detector for bayberry is composed of a light source module, light information acquisition module, sugar and acidity detection module, and detection result display module (OLED display). All modules are controlled by Arduino main control board (Figure 9), and the schematic diagram of the instrument is shown in Figure 10.

The following modules introduce the hardware of the instrument:

(1) Light source module: it is composed of LED1, LED2 and a blackout wall, which can remove the influence of ambient light and transmit specific wavelength light.
(2) Light information acquisition module: the light intensity sensor is used as the light information acquisition sensor to realize the function of converting the received light intensity signal into a digital signal.
(3) Sugar and acidity detection module: it is composed of the processor chip and the sugar and acidity detection model, which can convert the data received by the chip into the sugar and acidity result of the bayberry.
(4) Test result display module: it is composed of OLED display to realize the visualization function of bayberry test results.

#### 3.3.4. Instrument Control

**Figure 11:**
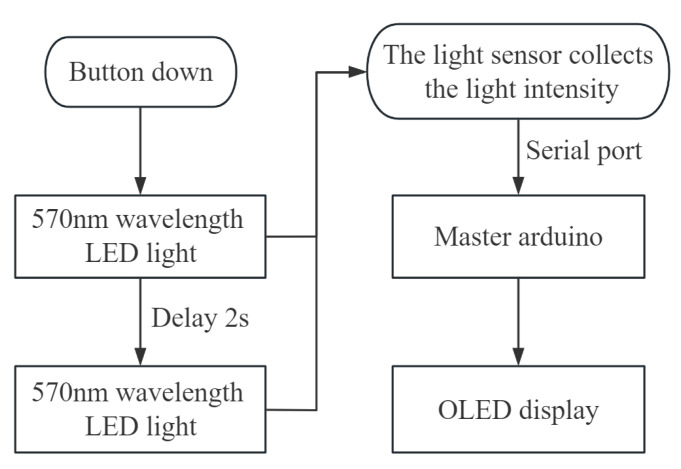
Portable bayberry quality detector control flow diagram.

The overall control of the instrument mainly includes the sequential switching control of two light sources of different wavelengths, the sensor data acquisition control synchronized with the light source switch, and the visual control of the detection results. The overall control flow diagram of the instrument is shown in Figure 11.

As shown in Figure 11, the Arduino main control board is controlled by touching the switch. After detecting that the key is closed once, the Arduino main control board will carry out the detection work. The specific control is as follows: First, power LED1 and power off after 1 s, and power LED2 for 1 s after 1 s. The data acquisition of the light intensity sensor is controlled according to the power switch of the two LED lights. When the two light sources are powered separately, the light intensity sensor will collect 1 s data synchronously and pass the collected reflected light data of the two light sources to the Arduino main control board, respectively. After receiving the two light data, the Arduino main control board will output the test results according to the calibration results and the sugar and acidity detection model. The two light dates are taken as the 570 nm reflection information and 610 nm reflection information. respectively, and in proportion to the calibration results of the two light sources to obtain the reflectivity information, which is further brought into the detection model and output to the display module. After receiving the information, the display module outputs it on the display screen, and the visual results include the sugar and acidity detection results of the bayberry fruit.

#### 3.3.5. Verification of Instrument Accuracy

**Figure 12:**
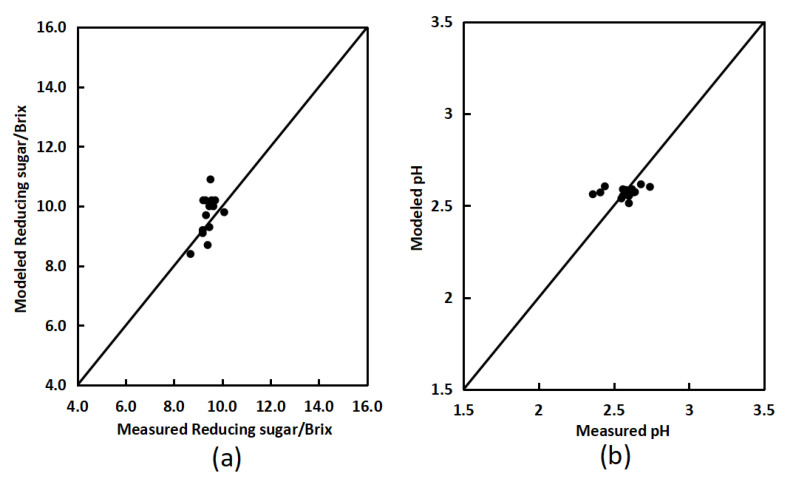
Comparison of modeled and measured values of reducing sugar (**a**) and pH (**b**).

A portable bayberry quality detector was used to test the sugar and acidity of fifteen bayberries. An average of the positive and negative detection results of each bayberry sample was calculated to reduce detection errors. The average of sugar and acidity was used as the predicted sugar and acidity value of the sample. The true values of pH and sugar content of bayberry samples were collected through the pH meter and the sugar meter as the measured sugar acidity values of the samples. The predicted and true values of sample sugar and acidity (Figure 12) were compared, calculating the standard deviation and accuracy of data, and the accuracy of the instrument (Equations (5) and (6)) was analyzed. The results are shown in Table 3.

(5) absolute deviation=|predicted value−ture value|

(6) $$accuracy=(1-\frac{mean\;absolute\;deviation}{average\;value})\;\times \;100$$

## 4. Conclusions

This article obtained hyperspectral data of bayberry fruit through experiments, measured the total sugar content, anthocyanin content, sugar content, pH value and other physicochemical parameters of bayberry fruit, and analyzed the experimental data. Based on correlation analysis, spectral models of various physicochemical parameters were established using a simple statistical regression model and utilizing sensitive bands spectral and spectral indices of bayberry, and the models were validated. Finally, the sugar acidity model of bayberry with better accuracy and stability was obtained. Based on this model, a low-cost portable the sugar and acidity detector of bayberry fruit was developed. The main conclusions of this paper are as follows:

(1) The spectra of bayberry fruits of different grades are clearly distinguished in the 530–630 nm wavelength range, and the reflectance decreases with the increase of bayberry quality. Therefore, the sensitive bands of bayberry fruit can be selected from the 530–630 nm wavelength range for modeling.
(2) The correlation between total sugar content, sugar content, pH value, and anthocyanins in bayberry fruit was 0.1449, 0.8098, and 0.6699, respectively. Only the sugar content and anthocyanins showed a good correlation, so a sugar content model can be established by inversion of anthocyanins using spectra and then conducting an inversion of anthocyanin using sugar content. Through experiments, it was found that the absorption peak positions of bayberry solutions shifted significantly at different pH levels, and as the pH value of the bayberry solution increased, the absorption peak of absorption spectrum of the bayberry solution showed a significant right shift trend. Therefore, a pH inversion model of bayberry can be established using spectroscopy.
(3) The normalization index composed of 620 nm and 630 nm, as well as the univariate quadratic model between the reflectance of 610 nm and anthocyanins, have the best effect, with the determination coefficients *R*^2^ of 0.6458 and 0.6795, respectively. Considering the development cost of the instrument, a 610 nm reflectance was selected to construct an anthocyanin inversion model. In the model of inverting sugar content with anthocyanins, the linear function relationship is the best, with the determination coefficient of 0.6658 and the verified RMSE of 1.339Brix. The normalization index composed of 620 nm and 630 nm, as well as the univariate quadratic model between reflectance of 570 nm and pH, have the best performance, with determination coefficients *R*^2^ of 0.7071 and 0.6589, respectively. Considering the development cost of the instrument, a 570 nm reflectance was selected to construct a pH inversion model.
(4) Based on the above model, a low-cost sugar acidity detector of bayberry was developed. Since the characteristic spectra and inversion results are consistent at low resolution and high resolution, the instrument uses a filter with 32 nm bandwidth, which greatly reduces the instrument cost. The software of this instrument includes feature band light sources, collection of lighting information, calculation spectral reflectance of feature band, and an inverse method of bayberry sugar and acidity. The hardware includes a light source module, a lighting information acquisition module, a sugar and acidity detection module, and a detection result display module (OLED display screen). In addition, the detection accuracy of the instrument was verified. The accuracy of sugar content and pH were 94.74% and 97.14%, respectively. The RRMSE values of sugar content and pH were 6.61% and 3.72%, respectively.

## Figures and Tables

**Table 1:** Component characteristics of different bayberry grades.

Physical and Chemical Parameters	Max	Min	Average	Standard Deviation
Anthocyanin (μg/g)	518.04	10.18	172.27	135.98
Total sugar (mg/g)	201.28	19.98	67.76	29.80
Sugar content (Brix)	13.50	5.41	8.18	1.59
pH	2.85	1.93	2.36	0.21

**Table 2:** Linear relationship, quadratic relationship, exponential relationship, logarithmic relationship and power function relationship between reflectance, normalized index NI and relative content of bayberry anthocyanin.

	Spectral Index	Function	$R^{2}$
Anthocyanin/(μg·g^−1^)	reflectance R (R610)	y = −15.02x + 370	0.6197
		y = 0.7375x^2^ − 36.21x + 483.7	0.6795
		y = 544.9 × exp(−0.1064 × x)	0.6776
		y = −410.5 × log10(x) + 600.6	0.6745
		y = −41,730 × x^0.004231^ + 42,330	0.6745
	NI (R620, R630)	y = −1978 × x + 27.42	0.6448
		y = −1831 × x^2^ − 2257 × x + 22.43	0.6458
		y = 70.36 × exp(−10.33 × x)	0.5978
pH	reflectance R (R570)	y = −0.02745x + 2.607	0.5687
		y = 0.001571x^2^ − 0.06363x + 2.73	0.6458
		y = 2.627 × exp(−0.01233 × x)	0.5841
		y = −0.5685 × log10(x) + 2.825	0.6541
		y = 1.515 × x^−0.2733^ + 1.434	0.6589
	NI (R620, R630)	y = −3.398 × x + 2.148	0.6930
		y = −11.45 × x^2^ − 5.092 × x + 2.122	0.7071
		y = 2.156 × exp(−1.403 × x)	0.6864

**Table 3:** Accuracy verification of bayberry reducing sugar and pH.

		Max	Min	Average	Accuracy
Sugar content /Brix	Modeled	10.080	8.683	9.419	94.74%
	Measured	10.900	8.400	9.729	
pH	Modeled	2.616	2.513	2.574	97.14%
	Measured	2.740	2.360	2.573	

## Data Availability

Data are contained within the article.
